# Fabrication and Characteristics of Chitosan Sponge as a Tissue Engineering Scaffold

**DOI:** 10.1155/2014/786892

**Published:** 2014-04-06

**Authors:** Takeshi Ikeda, Kahori Ikeda, Kouhei Yamamoto, Hidetaka Ishizaki, Yuu Yoshizawa, Kajiro Yanagiguchi, Shizuka Yamada, Yoshihiko Hayashi

**Affiliations:** Department of Cariology, Nagasaki University Graduate School of Biomedical Sciences, Nagasaki 852-8588, Japan

## Abstract

Cells, growth factors, and scaffolds are the three main factors required to create a tissue-engineered construct. After the appearance of bovine spongiform encephalopathy (BSE), considerable attention has therefore been focused on nonbovine materials. In this study, we examined the properties of a chitosan porous scaffold. A porous chitosan sponge was prepared by the controlled freezing and lyophilization of different concentrations of chitosan solutions. The materials were examined by scanning electron microscopy, and the porosity, tensile strength, and basic fibroblast growth factor (bFGF) release profiles from chitosan sponge were examined in vitro. The morphology of the chitosan scaffolds presented a typical microporous structure, with the pore size ranging from 50 to 200 **μ**m. The porosity of chitosan scaffolds with different concentrations was approximately 75–85%. A decreasing tendency for porosity was observed as the concentration of the chitosan increased. The relationship between the tensile properties and chitosan concentration indicated that the ultimate tensile strength for the sponge increased with a higher concentration. The in vitro bFGF release study showed that the higher the concentration of chitosan solution became, the longer the releasing time of the bFGF from the chitosan sponge was.

## 1. Introduction

Treatment strategies that induce the repair or regeneration of the loss or failure of tissues and organs by inducing cell proliferation and differentiation using tissue engineering methods constitute regenerative medicine [1]. When using such techniques, it is important to construct a suitable environment for the cell proliferation and differentiation, and the use of an effective combination of a scaffold with biomaterials for cell growth and bioactive molecules, such as cellular growth factors, is also indispensable. The biomaterials can be derived from natural sources [2–4], synthetic [5–8] polymers, and hybrid materials [9, 10] to generate a scaffold which often contains various growth factors or cell-surface interactive peptides to stimulate cell attachment, migration, and proliferation. A three-dimensional scaffold provides an extracellular matrix which functions as a necessary template for host cell infiltration and as a physical support to guide the cells' activity into the targeted tissues or organs. An ideal scaffold for tissue engineering should possess the characteristics of excellent biocompatibility, a suitable microstructure, controllable biodegradability, and good mechanical properties. In addition, a scaffold may be required to release bioactive substances at a controlled rate.

Generally, scaffolds are fabricated using variety of materials, including inorganic and organic polymers. The inorganic scaffolds include hydroxyapatite, *β*-tricalcium phosphate, and CaCO_3_[11–13] and the natural organic polymers include collagen, gelatin, cellulose, hyaluronic acid, and chitin or chitosan [14, 15].

Chitin exists in the exoskeleton of crustaceans, such as insects, crabs, and shrimps, as well as in the cell wall of bacilli [16, 17]. Chitosan is obtained by the alkaline deacetylation of chitin by industrial processing [18]. The ratio of glucosamine to the sum of glucosamine and N-acetylglucosamine is defined as the degree of deacetylation (DD) [19]. The average molecular weight (MW) of chitosan can range from 300 to 1,000 kDa with a DD of 30–95%, depending on the source and preparation procedure [20, 21]. The MW and DD are the predominant parameters that influence the solubility, mechanical strength, and degradation properties of chitosan and its derivatives.

A large amount of research concerning growth factors has already been conducted in the field of tissue regeneration, but there has not been a lot of success. It is thought that the major difficulty in obtaining the sustained release of growth factors is caused by the high diffusibility and the short half-life of growth factors themselves* in vivo*. For instance, when delivered without stabilization, basic fibroblast growth factor (bFGF) rapidly diffuses away from the implanted site, undergoes proteolysis, and consequently loses bioactivity under normal physiological conditions. It is therefore necessary to sustain its bioactivity in order to increase the utility of bFGF in practical treatments by developing dependable controlled release systems.

It has been known that various growth factors contribute to tissue regeneration for cell proliferation and differentiation. The bFGF, a 17-kDa polypeptide, is a multifunctional protein that promotes angiogenesis and regulates many aspects of cellular activity, including cell proliferation, migration, and ECM metabolism, in a time- and concentration-dependent manner [22–25]. Furthermore, it has been reported that bFGF can exert a significant effect on the cell proliferation of human dental pulp stem cell isolated by immunomagnetic bead selection method for STRO-1 and that among the cytokines and growth factors, bFGF is clinically proven, having demonstrated accelerated acute and chronic wound healing [26].

When a chitosan containing bFGF was applied for wound healing in db/db mice, a significant effect was shown on granulation tissue formation, infiltrating cells, and the capillary number, but only a minor effect was observed on the degree of reepithelialization [27]. However, little is known about how a growth factor is released from a fabricated chitosan sponge in the tissue regeneration. The purpose of the present study was to investigate the microstructural characteristics, the mechanical properties, and the bFGF-releasing kinetics of chitosan sponge as a tissue engineering scaffold.

## 2. Material and Methods

### 2.1. Chitosan Scaffold Fabrication

Chitosan polymer (MW = 100,000 Da, degree of deacetylation = 85%) was supplied by Koyo Chemical Co. (Osaka, Japan). Chitosan scaffolds were prepared by a thermally-induced phase separation method. Chitosan solutions with concentrations of 1, 2, or 4 wt% were prepared by dissolving the chitosan in 2 wt% acetic acid. This solution was stirred gently for 2~5 h at 50°C until it became transparent and was neutralized to pH 7.4 using 1 N NaOH, and the air remaining in solution was removed by a vacuum pump for 24 h. Bulk chitosan scaffold samples were prepared by freezing and lyophilizing chitosan solutions in a precooled 96-well plate. The resulting cylindrical sample was 6 mm in diameter and 10 mm in height and was accomplished using 0.4 mL of the above solution per well, followed by refrigeration at 4°C for 2 h. The subsequent cooling schedule was at 0°C for 18 h and −35°C for 24 h, followed by −80°C for 24 h. Finally, the chitosan solution was completely lyophilized by freeze-drying at −80°C for 72 h [28].

### 2.2. Microstructural Characteristics

To observe the surface and internal microstructure of the chitosan sponge, the freeze-dried samples were mounted on aluminum holders and coated with carbon using a vacuum evaporator. Their pore structures were examined using a Hitachi S-3500 scanning electron microscope (Hitachi Ltd, Tokyo, Japan) operating at 20 kV. The mean pore diameters were estimated from scanning electron micrographs by measuring 100 different pores for each scaffold using a computed image analyzer [29]. A comparative study was carried out on the pore diameter and porosity in the sponges made with different concentrations of chitosan solution.

### 2.3. Measurements of Porosity

A liquid displacement method was used to measure the porosity of the scaffolds [30, 31]. A scaffold of weight *W* was immersed into a graduated cylinder containing a known volume (*V*_1_) of ethanol. The cylinder was placed in a vacuum to force the ethanol into the pores of the scaffold until no air bubbles were observed emerging from the scaffold. The total volume of the ethanol and scaffold was recorded as *V*_2_. The volume difference (*V*_2_ − *V*_1_) was the volume of the skeleton of the scaffold. The scaffold was removed from the ethanol and the residual ethanol volume was measured as *V*_3_. The quantity (*V*_1_ − *V*_3_) was the volume of the ethanol impregnated in the scaffold and was considered to be the void volume of the scaffold. The total volume (*V*) of the scaffold was thus determined as *V* = (*V*_2_ − *V*_1_) + (*V*_1_ − *V*_3_) = *V*_2_ − *V*_3_. The porosity (*ε*) of the scaffold was evaluated as *ε* = (*V*_1_ − *V*_3_)/(*V*_2_ − *V*_3_). Replicate measurements were made, and the results are reported as average values.

### 2.4. Mechanical Properties

The ultimate tensile strength of the scaffolds in the wet state was measured by a universal testing machine (AG-X 50N, SHIMADZU, Kyoto, Japan) according to procedures outlined in the ASTMD 3574-E at room temperature. The three-dimensional cylindrical scaffold was thoroughly hydrated with a 0.1 M PBS solution while mounted on the grip and was pulled to break at a constant cross head speed of 1 mm/min under 65% relative humidity until failure [32]. A tensile stress-strain curve was generated from each sample, and Young's modulus was calculated by drawing a tangent to the initial linear portion of the stress-strain curve. The average and standard deviations of measurements from five samples are reported [33].

### 2.5. *In Vitro* bFGF Release Study

FIBLAST spray 250 (Kaken Pharmaceutical co., Tokyo, Japan), made from rh-bFGF, has already been validated as a cell growth factor and was added to the sponge body. The release kinetics of bFGF from the chitosan sponge was monitored for 28 days after the sponge had been soaked in PBS (pH 7.4). The bFGF release profiles from the chitosan scaffold of the three different concentrations and a gelatin scaffold (MedGel, Wako Pure Chemical Industries, Ltd., Osaka, Japan) used as a control were determined* in vitro* by examining the concentration of bFGF and were quantitatively measured using an enzyme-linked immunosorbent assay (ELISA) kit (Human FGF basic Immunoassay, catalog number DFB50, R&D Systems, Minneapolis, MN) according to the manufacturer's protocol. Briefly, the samples were dipped in 5.0 mL sterile PBS solution and kept in a shaking incubator (37°C, 40 rpm) for various time periods up to four weeks. At the designated time points at three, six, and 12 hours, and one, three, seven, 14, 21, and 28 days, the supernatant was collected and an equal amount of fresh medium was added to each sample. A total of 100 *μ*L/well of 2 *μ*g/mL anti-bFGF as the capture antibody was immobilized on a 96-well high-binding polystyrene plate (NUNC, Denmark) by incubation at room temperature for 18 h. The plate wells were then blocked with 200 *μ*L/well DPBS, including 1% BSA, 5% sucrose, and 0.05% NaN_3_ for 1 h. A total of 100 *μ*L/well of bFGF solution was added to the plate, and the plate was incubated for 1 h at room temperature. At the same time, a dilution series of known concentrations of human bFGF was prepared on each plate to serve as the standard. Next, 100 *μ*L/well of biotin-anti-bFGF (0.5 *μ*g/mL) was added as the detection antibody, and the plate was incubated for another hour. The plate wells were then emptied and washed three times with 200 *μ*L/well wash buffer (DPBS with 0.05% Tween-20, pH 7.4), followed by the addition of 100 *μ*L/well of a 1 : 1000 dilution of streptavidin-horseradish peroxidase. After 1 h incubation at room temperature, each well was loaded with 100 *μ*L of TMB, and a blue color gradually appeared. The wells were washed again, after which the color-forming reaction was stopped by the addition of 100 *μ*L/well of 1 M sulfuric acid. Finally, the plate was read at 450 nm on a microplate reader (BIO-RAD 550, Bio-Rad Laboratories, California, USA) to determine the bFGF level released, with the standard bFGF samples providing the data for calibration. A calibration curve was obtained using a standard bFGF solution of a known concentration. The accumulated bFGF quantities released during the different time periods were then calculated.

### 2.6. Statistical Analysis

Experiments were run in triplicate per sample, and all data were expressed as the means ± standard deviation (SD). Statistical analyses of the data were performed using a one-way analysis of variance (ANOVA). The difference was regarded to be statistically significant for values of *P* < 0.05.

## 3. Results and Discussion

The use of materials of mammalian origin in medical treatment is being greatly limited after the appearance of BSE. Based on this background of high concern about the medical safety of biomaterials, it is expected that the application of a native organic physiological material that cannot support the transmission of BSE or similar pathologies will be very meaningful in the tissue engineering field. This viewpoint supports the use of chitin and its derivatives as candidate scaffolds. Furthermore, the investigation of functional biomaterials has been directed towards the development of improved scaffolds and better controlled-release drug delivery systems.

### 3.1. Characteristics of the Chitosan Sponge

**Figure 1:**
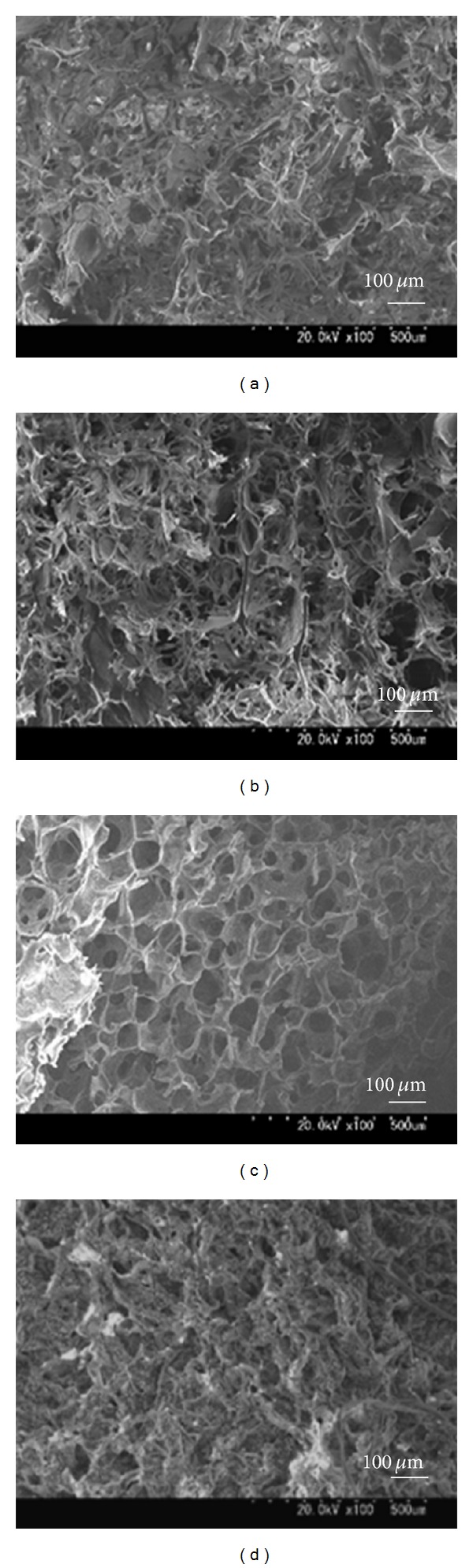
Cross-sectional SEM images of chitosan scaffolds (CS) made with different concentrations. (×100) (a): 1 wt% CS; (b): 2 wt% CS; (c): 4 wt% CS; (d): MedGel (containing gelatin, used as a control).

Figure 1 illustrates the scanning electron microscopic (SEM) view of each sponge. The morphology of the chitosan scaffolds presented a typical microstructure of polymeric foam prepared by thermally-induced phase separation. The surface appearance of each sponge did not differ significantly based on the different concentrations of the chitosan solution except the 4 wt% chitosan, which had clearly porous form and the pores were interconnected. The average diameter of the pores was 158.5 *μ*m for the 1 wt% chitosan sponge, 142.5 *μ*m for the 2 wt% chitosan, and 74.5 *μ*m for the 4 wt% chitosan. In the gelatin sponge (MedGel) used as a control, the average pore size was 138.5 *μ*m (Figure 3). There were significant differences between the 4 wt% chitosan and each of the other concentrations and gelatin. In our present studies, it was revealed that as the concentration of the chitosan increased, the pore wall tended to become thicker and more homogenous and the surface morphology also became more dense (Figure 1).

**Figure 2:**
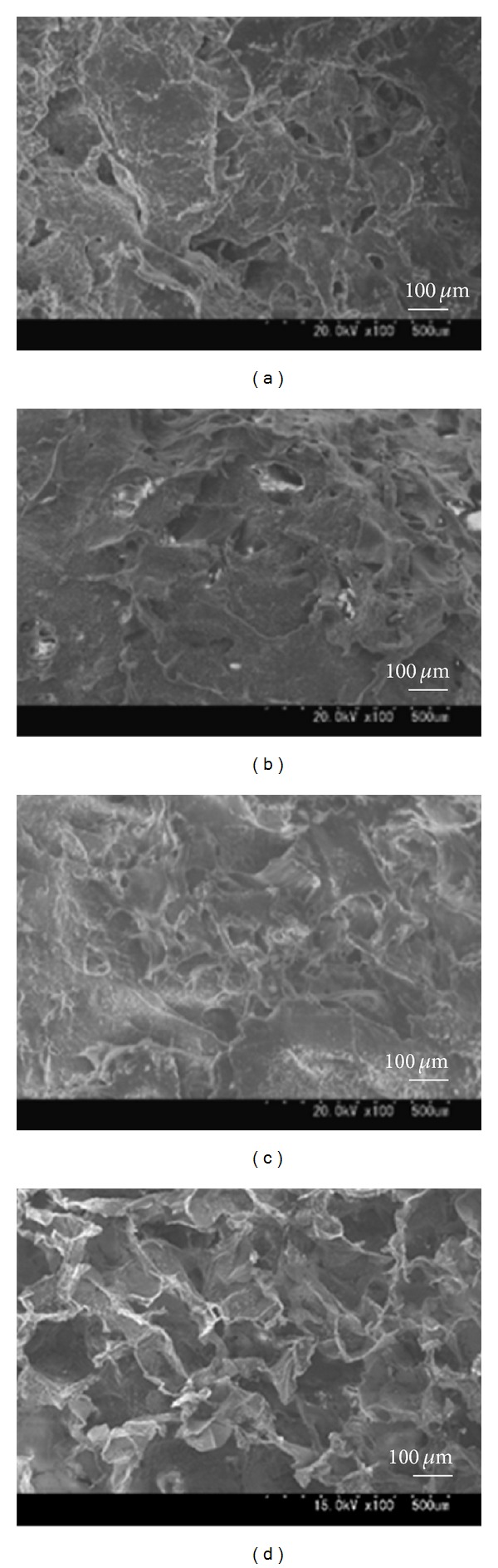
Cross-sectional SEM images of chitosan scaffolds (CS) made with different concentrations after incubation in a PBS solution at 37°C for 4 weeks (×100). (a): 1 wt% CS; (b): 2 wt% CS; (c): 4 wt% CS; (d): MedGel (containing gelatin, used as a control).

**Figure 3:**
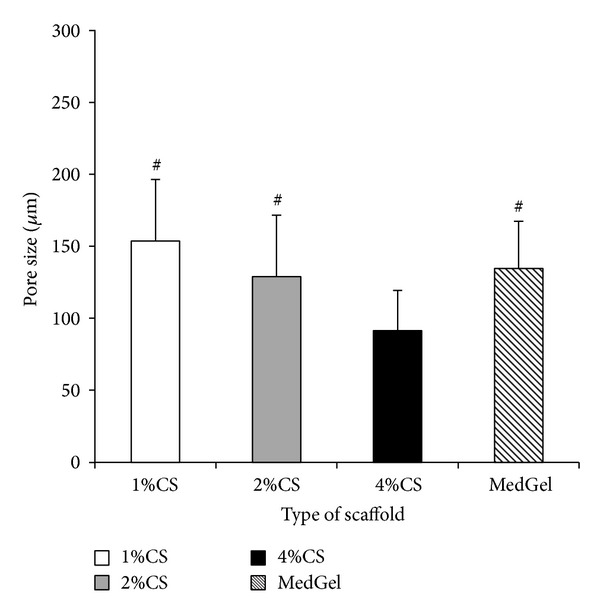
Comparison of the mean pore size of each sponge. The data are presented as the means ± SD (*n* = 5). ^#^ *P* < 0.01 compared with 4 wt% chitosan solution (CS).

It was reported that there are no significant differences in the cell infiltration into sponges with pore sizes ranging from 50 to 200 *μ*m [34]. From this perspective, the pore size of our chitosan sponges is acceptable. The SEM images taken 28 days after soaking the sponges in PBS demonstrated that although a collapse of the pore shape was prominent in the 1 wt% and 2 wt% chitosan groups, there was improved stability in the 4 wt% chitosan group and the gelatin sponge (Figure 2).

**Figure 4:**
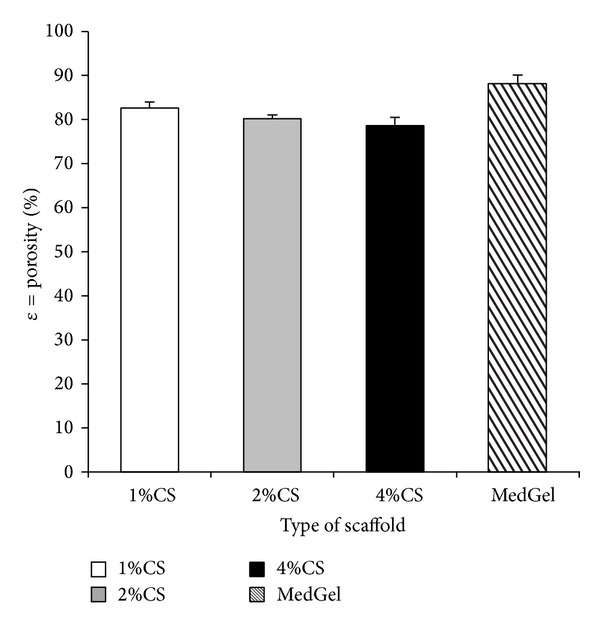
Comparison of the porosity of each sponge type. The data are presented as the means ± SD (*n* = 5).

The porosity of chitosan scaffolds with different concentrations is shown in Figure 4. It was observed that the porosity of the 1 wt% chitosan group was 82.6%, while that of the 2 wt% chitosan group was 80.2% and that of the 4 wt% chitosan was 78.6% and that of the gelatin was 88.1%. A decreasing trend in the porosity was observed as the concentration of the chitosan increased, although it was always approximately 75–85%, and no significant differences were observed between the groups.

The porosity is an important parameter for the tissue engineering scaffolds. Scaffolds must have sufficient porosity for nutrient and gas exchange. It has been previously reported that a porosity of more than 80% was characteristic of an ideal scaffold [35, 36]. In the present study, the 1 wt% and 2 wt% chitosan scaffolds met this criterion. Many other studies have indicated that a decreased pore size and increased thickness of the pore wall can result in higher tensile and compressive strength [37]. This provides evidence that the mechanical properties of chitosan porous scaffolds can be improved by increasing the chitosan concentration. However, if the chitosan concentration was increased up to 10 wt%, the chitosan solution tended to aggregate to form larger clusters due to the increased viscosity of the mixture (personal communication). Consequently, the aggregates caused the porosity to decrease dramatically. Moreover, it was suggested that changes in porosity also affected the mechanical properties of the scaffold. The chitosan scaffold is sufficiently porous, so that it can provide a large area of internal surface for cell adhesion and migration and can also make it easy for the exchange of nutrients and metabolic waste.

### 3.2. Mechanical Properties

The mechanical properties of a scaffold used for tissue engineering are very important due to the need for the structural stability to oppose the various stresses incurred during culture* in vitro* or implantation* in vivo*. The mechanical properties of the different concentrations of chitosan solution were evaluated by tensile strength measurements. The tensile strength values were 0.07 ± 0.01, 0.12 ± 0.03, and 0.44 ± 0.08 MPa for 1 wt%, 2 wt%, and 4 wt%, respectively. The tensile strength of the gelatin sponge used as a control was 0.03 ± 0.01 MPa. These results strongly indicated that the tensile strength of the 2 wt% and 4 wt% chitosan sponges was significantly higher compared to that of gelatin sponge. The relationship between the tensile properties and chitosan concentration is summarized in Table 1. The ultimate tensile strength of the sponge increased as the concentration of the solution increased. It is well known that the tensile strengths of porous structures have been reported to be in the range of 0.03–0.06 MPa [38, 39] and Lin et al. also reported that the porosity would influence the mechanical properties of porous scaffolds [40]. Our results (Table 1) suggest that both the tensile strength and Young's modulus decrease progressively with the increase in porosity of the scaffolds, which is fundamentally in agreement with other results [41, 42]. It has been reported that the pore size also affects the mechanical properties of porous scaffolds, and the foams created with the smaller pore sizes have weaker compressive mechanical properties compared to the foams with larger pores [43, 44]. The pore size of scaffolds could be regulated by altering the freezing temperature, but the porosity of scaffolds did not follow this change. By optimizing the processing conditions, interconnected porous structures with well controlled porosities and pore sizes for desirable scaffolds may be achieved.

The mechanical stability of the scaffolds for tissue engineering is necessary to maintain the cell differentiation and proliferation by withstanding various stresses incurred during implantation* in vivo* and culture* in vitro*. Thus, sufficient mechanical properties of the scaffolds are very important to survive living cells and to provide appropriate physical support in case of nervous and vascular defects [45–48]. Therefore, the tensile strength and Young's modulus of 4 wt% chitosan sponge were sufficient enough for achieving these purposes.

### 3.3. *In Vitro* Release Kinetics

**Figure 5:**
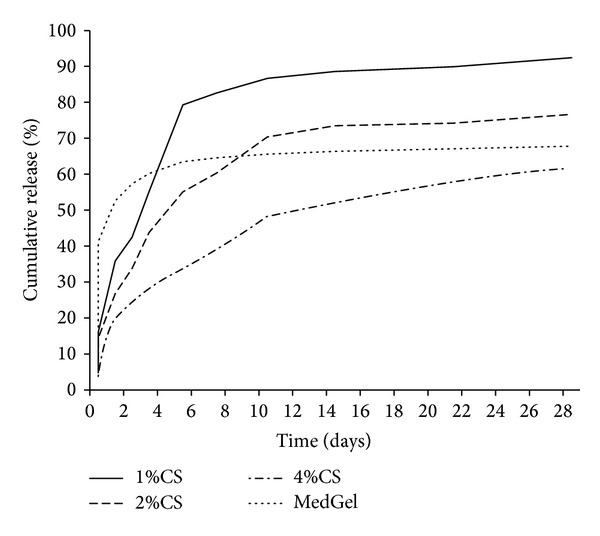
The bFGF release profiles from the chitosan sponges with different concentrations and from the gelatin scaffold.

When we examined the release kinetics of bFGF from the chitosan sponge, approximately 80% was separated in the 1 wt% chitosan group by one week after being soaked, and about 70% was separated in the 2 wt% chitosan group after two weeks had passed, while 50% was still present in the 4 wt% chitosan group after two weeks had passed, and it continued to be released thereafter (Figure 5). It was noted that as the concentration of the chitosan solutions increased, the release of the bFGF contained in the chitosan sponge decreased. These findings indicate that the 4 wt% chitosan sponge can function as a scaffold to provide the sustained release of bFGF. This suggests that since the amount of growth factor bound to the chitosan molecule through ionic interactions by combining with oppositely charged chitosan and bFGF had increased, the release period tended to be prolonged by an electrostatic effect [49].

However, it has generally been thought that the direct supply of growth factors in soluble form into a regeneration site is not effective [50]. Many kinds of growth factors rapidly diffuse away from the application site. To enable growth factors to efficiently exert their biological effects in the body, it is necessary to ensure that there is a controlled release of the growth factor to the application site over an extended time period. This can be made possible by incorporating the factor into an appropriate scaffold. It is likely that such scaffold will also protect the growth factor against proteolysis, allowing it to have prolonged retention of activity* in vivo*.

Another important factor is that the scaffold should be gradually degraded in the body, because it is not needed any more after the growth factor release has been completed. Thus, the scaffold used for tissue regeneration or organ substitution should possess properties that allow it to create an environment suitable for the induction of tissue regeneration by making use of biodegradability and drug delivery technology.

We previously reported an investigation of the biodegradation processes of chitosan fibers implanted in the bone tissue and found that it should be a suitable biomaterial for bone surgery and bone regenerative medicine [51]. It was suggested that chitosan -based materials bind to growth factors and release them in a controlled manner due to their cationic nature and predictable degradation rate.

## 4. Conclusions

The present findings indicate that the fabricated chitosan sponge has excellent biomechanical properties and may be a preferable candidate scaffold, since it can also effectively serve as a carrier of growth factors because of its structural, mechanical, and releasing properties.

## Figures and Tables

**Table 1:** The mechanical properties of sponges with different concentrations of chitosan in the wet state (average ± standard deviation of at least five samples).

Type of sponge	Tensile strength (Mpa)	Young's modulus (Mpa)
1 wt%	0.07 ± 0.01^Ab^	0.05 ± 0.02^Bb^
2 wt%	0.12 ± 0.03^Ac^	0.23 ± 0.06^Bc^
4 wt%	0.44 ± 0.08^Ad^	2.02 ± 0.11^Bd^
Gelatin	0.03 ± 0.01^Aa^	0.02 ± 0.02^Ba^

Different superscript letters indicate statistically significant differences between groups (*P* < 0.05).

## References

[1] Langer R, Vacanti JP (1993). Tissue engineering. *Science*.

[2] Livesey SA, Herndon DN, Hollyoak MA, Atkinson YH, Nag A (1995). Transplanted acellular allograft dermal matrix: potential as a template for the reconstruction of viable dermis. *Transplantation*.

[3] Black AF, Berthod F, L’Heureux N, Germain L, Auger FA (1998). In vitro reconstruction of a human capillary-like network in a tissue- engineered skin equivalent. *The FASEB Journal*.

[4] Badylak S, Liang A, Record R, Tullius R, Jason Hodde JH (1999). Endothelial cell adherence to small intestinal submucosa: an acellular bioscaffold. *Biomaterials*.

[5] Kim BS, Mooney DJ (1998). Development of biocompatible synthetic extracellular matrices for tissue engineering. *Trends in Biotechnology*.

[6] Oberpenning F, Meng J, Yoo JJ, Atala A (1999). De novo reconstitution of a functional mammalian urinary bladder by tissue engineering. *Nature Biotechnology*.

[7] Ferber D (1999). Lab-grown organs begin to take shape. *Science*.

[8] Niklason LE, Gao J, Abbott WM (1999). Functional arteries grown in vitro. *Science*.

[9] Shakesheff KM, Cannizzaro SM, Langer R (1998). Creating biomimetic micro-environments with synthetic polymer-peptide hybrid molecules. *Journal of Biomaterials Science, Polymer Edition*.

[10] Patel N, Padera R, Sanders GHW (1998). Spatially controlled cell engineering on biodegradable polymer surfaces. *The FASEB Journal*.

[11] Porter JR, Ruckh TT, Popat KC (2009). Bone tissue engineering: a review in bone biomimetics and drug delivery strategies. *Biotechnology Progress*.

[12] Cho YR, Gosain AK (2004). Biomaterials in craniofacial reconstruction. *Clinics in Plastic Surgery*.

[13] Kretlow JD, Young S, Klouda L, Wong M, Mikos AG (2009). Injectable biomaterials for regenerating complex craniofacial tissues. *Advanced Materials*.

[14] Liu X, Ma L, Mao Z, Gao C (2011). Chitosan-based biomaterials for tissue repair and regeneration. *Advances in Polymer Science*.

[15] Khan F, Ahmad SR (2013). Polysaccharides and their derivatives for versatile tissue engineering application. *Macromolecular Bioscience*.

[16] Giraud-Guille M-M (1984). Fine structure of the chitin-protein system in the crab cuticle. *Tissue and Cell*.

[17] Muzzarelli RA (1973). Chitosan. *Natural Chelating Polymers*.

[18] Borzacchiello A, Ambrosio L, Netti PA (2001). Chitosan-based hydrogels: synthesis and characterization. *Journal of Materials Science: Materials in Medicine*.

[19] Hirano S, Tsuchida H, Nagao N (1989). N-acetylation in chitosan and the rate of its enzymic hydrolysis. *Biomaterials*.

[20] Nosé Y, DeBakey ME (2001). Historical perspectives of hybrid hepatic assist devices: tissue sourcing, immunoisolation, and clinical trial. *Annals of the New York Academy of Sciences*.

[21] Vandevord PJ, Matthew HWT, Desilva SP, Mayton L, Wu B, Wooley PH (2002). Evaluation of the biocompatibility of a chitosan scaffold in mice. *Journal of Biomedical Materials Research*.

[22] Dereka XE, Markopoulou CE, Mamalis A, Pepelassi E, Vrotsos IA (2006). Time- and dose-dependent mitogenic effect of basic fibroblast growth factor combined with different bone graft materials: an in vitro study. *Clinical Oral Implants Research*.

[23] Rifkin DB, Moscatelli D (1989). Recent developments in the cell biology of basic fibroblast growth factor. *Journal of Cell Biology*.

[24] Tsuboi R, Rifkin DB (1990). Recombinant basic fibroblast growth factor stimulates wound healing in healing-impaired db/db mice. *Journal of Experimental Medicine*.

[25] Jackson CL, Reidy MA (1993). Basic fibroblast growth factor: Its role in the control of smooth muscle cell migration. *The American Journal of Pathology*.

[26] He H, Yu J, Liu Y (2008). Effects of FGF2 and TGF*β*1 on the differentiation of human dental pulp stem cells in vitro. *Cell Biology International*.

[27] Obara K, Ishihara M, Ishizuka T (2003). Photocrosslinkable chitosan hydrogel containing fibroblast growth factor-2 stimulates wound healing in healing-impaired db/db mice. *Biomaterials*.

[28] Ho MH, Kuo PY, Hsieh HJ (2004). Preparation of porous scaffolds by using freeze-extraction and freeze-gelation methods. *Biomaterials*.

[29] Wu J, Liao C, Zhang J (2011). Incorporation of protein-loaded microspheres into chitosan-polycaprolactone scaffolds for controlled release. *Carbohydrate Polymers*.

[30] Nazarov R, Jin HJ, Kaplan DL (2004). Porous 3-D scaffolds from regenerated silk fibroin. *Biomacromolecules*.

[31] Zhang RY, Ma PX (1999). Poly (*α*-hydroxyl acids)/hydroxyapatite porous composites for bone-tissue engineering. I. Preparation and morphology. *Journal of Biomedical Material Research*.

[32] Lefler A, Ghanem A (2009). Development of bFGF-chitosan matrices and their interactions with human dermal fibroblast cells. *Journal of Biomaterials Science, Polymer Edition*.

[33] Kim SE, Cho YW, Kang EJ (2001). Three-dimensional porous collagen/chitosan complex sponge for tissue engineering. *Fibers and Polymers*.

[34] Dawlee S, Sugandhi A, Balakrishnan B, Labarre D, Jayakrishnan A (2005). Oxidized chondroitin sulfate-cross-linked gelatin matrixes: a new class of hydrogels. *Biomacromolecules*.

[35] Attawia MA, Herbert KM, Uhrich KE, Langer R, Laurencin CT (1999). Proliferation, morphology, and protein expression by osteoblasts cultured on poly (anhydride-co-imides). *Journal of Biomedical Materials Research*.

[36] She Z, Zhang B, Jin C, Feng Q, Xu Y (2008). Preparation and in vitro degradation of porous three-dimensional silk fibroin/chitosan scaffold. *Polymer Degradation and Stability*.

[37] Kim U-J, Park J, Joo Kim H, Wada M, Kaplan DL (2005). Three-dimensional aqueous-derived biomaterial scaffolds from silk fibroin. *Biomaterials*.

[38] Madihally SV, Matthew HWT (1999). Porous chitosan scaffolds for tissue engineering. *Biomaterials*.

[39] Francis Suh JK, Matthew HWT (2000). Application of chitosan-based polysaccharide biomaterials in cartilage tissue engineering: a review. *Biomaterials*.

[40] Lin ASP, Barrows TH, Cartmell SH, Guldberg RE (2003). Microarchitectural and mechanical characterization of oriented porous polymer scaffolds. *Biomaterials*.

[41] Lee SB, Kim YH, Chong MS, Lee YM (2004). Preparation and characteristics of hybrid scaffolds composed of *β*-chitin and collagen. *Biomaterials*.

[42] Tu C, Cai Q, Yang J, Wan Y, Bei J, Wang S (2003). The fabrication and characterization of poly(lactic acid) scaffolds for tissue engineering by improved solid-liquid phase separation. *Polymers for Advanced Technologies*.

[43] Ma PX, Choi J-W (2001). Biodegradable polymer scaffolds with well-defined interconnected spherical pore network. *Tissue Engineering*.

[44] Chen VJ, Ma PX (2004). Nano-fibrous poly(L-lactic acid) scaffolds with interconnected spherical macropores. *Biomaterials*.

[45] Talini KH, Geuna S, Dahlin LB (2013). Chitosan tubes of varying degrees of acetylation for bridging peripheral nerve defects. *Biomaterials*.

[46] Gu Y, Zhu J, Xue C (2014). Chitosan/silk fibroin-based, Schwann cell-derived extracellular matrix-modified scaffolds for bridging rat sciatic nerve gaps. *Biomaterials*.

[47] Bačáková L, Novotná K, Pařízek M (2014). Polysaccharides as cell carriers for tissue engineering: the use of cellulose in vascular wall reconstruction. *Physiological Research*.

[48] Min Z, Wei Q, Zhao L (2014). Development and *in vivo* evaluation of small-diameter vascular grafts engineered by outgrowth endothelial cells and electrospun chitosan/poly(*ε*-caprolactone) nanofibrous scaffolds. *Tissue Engineering A*.

[49] Rinaudo M (2008). Main properties and current applications of some polysaccharides as biomaterials. *Polymer International*.

[50] Tabata Y (2003). Tissue regeneration based on growth factor release. *Tissue Engineering*.

[51] Ikeda T, Yanagiguchi K, Matsunaga T (2005). Immunohistochemical and electron microscopic study of the biodegradation processes of chitin and chitosan implanted in rat alveolar bone. *Oral Medicine and Pathology*.

